# Beyond fluorescence-guided resection: 5-ALA-based glioblastoma therapies

**DOI:** 10.1007/s00701-024-06049-3

**Published:** 2024-04-02

**Authors:** Walter Stummer, Michael Müther, Dorothee Spille

**Affiliations:** Department of Neurosurgery, University Hospital Münster, Albert-Schweitzer-Campus 1, Building A1, 48149 Münster, Germany

**Keywords:** Glioblastoma, Reactive oxygen species, Aminolevulinic acid, Photosensitizing agents

## Abstract

Glioblastoma is the most common primary malignant brain tumor. Despite advances in multimodal concepts over the last decades, prognosis remains poor. Treatment of patients with glioblastoma remains a considerable challenge due to the infiltrative nature of the tumor, rapid growth rates, and tumor heterogeneity. Standard therapy consists of maximally safe microsurgical resection followed by adjuvant radio- and chemotherapy with temozolomide. In recent years, local therapies have been extensively investigated in experimental as well as translational levels. External stimuli-responsive therapies such as Photodynamic Therapy (PDT), Sonodynamic Therapy (SDT) and Radiodynamic Therapy (RDT) can induce cell death mechanisms via generation of reactive oxygen species (ROS) after administration of five-aminolevulinic acid (5-ALA), which induces the formation of sensitizing porphyrins within tumor tissue. Preliminary data from clinical trials are available. The aim of this review is to summarize the status of such therapeutic approaches as an adjunct to current standard therapy in glioblastoma.

## Introduction

Glioblastoma is the most common malignant brain tumor [25]. Current standard treatment compromises maximally safe tumor resection with adjuvant concomitant stereotactic fractionated radiotherapy and chemotherapy with temozolomide followed by six cycles of adjuvant temozolomide maintenance [46]. Even though median survival times of up to 30 months can be reached [28], recurrence rates are high [25, 47]. Supramarginal resection as a significant predictor for increased OS and PFS is not always feasible due functional limits of the resection [2, 36]. Radiation therapy carries the risk of neurocognitive decline and development of radiation necrosis [5]. Key challenges in systemic therapies for patients with glioblastoma include intratumoral heterogeneity, immunosuppressive tumor microenvironment, and the blood–brain barrier [42]. To overcome the abovementioned hurdles, it is desirable to apply local tumor therapies that address the neoplastic lesion directly at an overall low level of toxicity.

Five-aminolevulinic acid (5-ALA) is a prodrug that is administered orally and converted into protoporphyrin IX (PPIX) in mitochondria. In malignant glioma, PPIX accumulates and can be visualized during surgery with appropriate hardware [35]. Fluorescence-guided resection using 5-ALA is considered guideline-based standard in surgical treatment of malignant glioma [46].

**Fig. 1:**
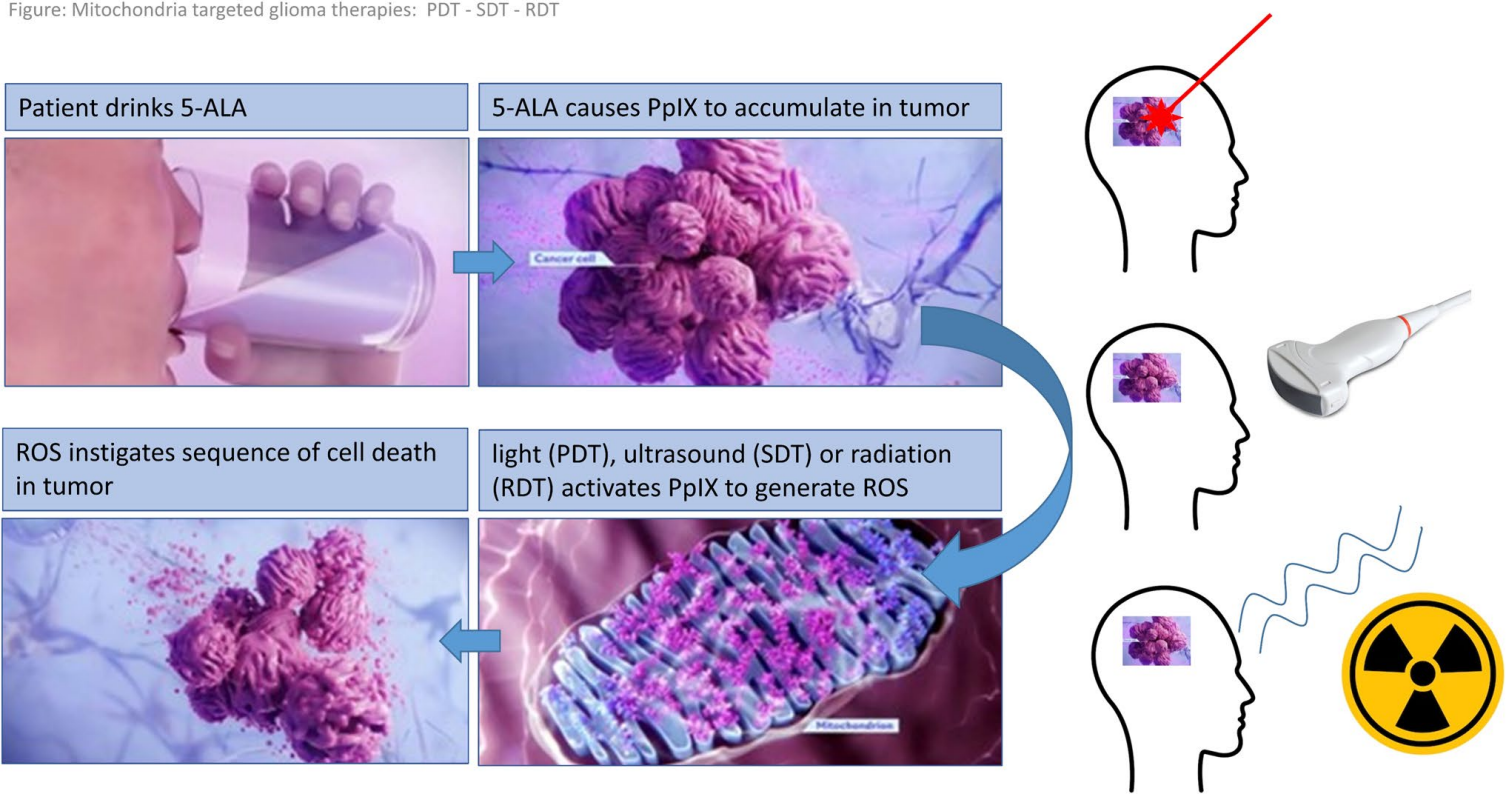
Mitochondria-targeted glioma therapies: PDT—SDT—RDT

5-ALA not only serves as an agent for inducing tissue fluorescence. 5-ALA derived tumor porphyrins, foremost PPIX appear to have a potential as sensitizers to render the tumor more susceptible to certain external stimuli. After treatment with light, sound, radiation, or magnetism, reactive oxygen species (ROS) can be locally generated that further induce cell death mechanisms (depicted in Fig. 1). These so-called external stimuli-responsive therapies have steadily raised interest in the treatment of glioblastoma in recent years and show promising antitumor effects [44]. In particular, photodynamic therapy (PDT) is already used in clinical practice in the treatment of patients with glioblastoma using 5-ALA, a drug already approved by FDA and EMA for fluorescence-guided resections [8] [29, 43].

5-ALA-induced porphyrins are possibly very attractive targets as they cannot only be found in tumor cells but also in myeloid cells, microglia, and T cells in the tumor mass, these cells supporting a pro-tumor, antiangiogenic microenvironment [14, 19, 23].

The aim of this review is to provide an overview of the possibilities and current clinical trial landscape of 5-ALA-PPIX-based therapies in the treatment of glioblastoma.

## Search criteria

A comprehensive search was performed in PubMed, Embase, Web of Science, and the Cochrane Central Register of Controlled Trials in October 2023. The following medial subject headings were applied: “photodynamic therapy” OR “sonodynamic therapy” OR “radiodynamic therapy”” AND (“5-ALA” OR “ALA” OR “PpIX”) AND “glioma” OR “glioblastoma” OR “astrocytoma.” The trial registry ClinicalTrials.gov was searched to identify ongoing clinical trials.

## Photodynamic therapy (PDT)

Photodynamic therapy is a procedure based on the increased production of ROS in tumorous tissue. An increased release of ROS causes oxidative stress and corresponding cell damage. ROS-generating photosensitizers in general are externally stimulated with light (photodynamic therapy, PDT), ultrasound (sonodynamic therapy, SDT), ionizing radiation (radiodynamic therapy, RDT), microwaves (microwave dynamic therapy, MDT), or alternating current (electrodynamic therapy, EDT) [48, 50].

Photodynamic therapy is a noninvasive procedure that has found its way into the treatment of oncological (breast, bladder, and esophageal cancer as well as glioblastoma) and non-oncological diseases in recent years [49]. PDT involves the topical or intravenous application of a photosensitizer, which accumulates specifically in tumor tissue. Light of specific wavelength (between 600 and 850 nm) is then applied. This in turn leads to excitation of the photosensitizer with consecutive conversion of the light energy into the generation of ROS and oxygen radicals. This process is promoted by addition of molecular oxygen and results in cell damage [8].

In the field of glioma surgery, the procedure is being studied in the intraoperative treatment of malignant gliomas and brain metastases [1, 21, 22, 29, 34, 37, 51]. On the one hand, the method of stereotactic, interstitial PDT was described. Instead of an open surgical procedure, cylindrical light diffusers are inserted into the tumor and light is emitted [3, 17, 27].

In the brain, light penetration poses a major challenge to the volume of tumor that can be treated [3]. Typically, with 5-ALA-induced porphyrins, and choosing the longest possible wavelength for activating porphyrins of 635 nm, clinically useful penetration depths within an acceptable period of illumination of 1 h, without critically increasing tissue temperatures, are only 4–5 mm [3]. Thus, in many tumors multiple, stereotactically implanted light diffusors become necessary.

On the other hand, an open form of photodynamic therapy has been described, in which resection is followed by light irradiation of resection cavities. This offers the possibility of treating tumor mass or infiltrating cells that have to be left behind for functional reasons [10, 11, 21, 29]. The treatment is intended for unifocal lesions with a limited tumor volume, as tissues need to be penetrated by light. Also, treating larger tumor masses carries the risk of developing expansive cytotoxic edema [18].

In general, PDT is believed to have a synergistic effect to other forms of therapy, due to the fact that the blood–brain barrier becomes more permeable [32]. In addition, local inflammatory processes with increased recruitment of antigen-presenting cells have been demonstrated in in vitro studies [6, 20]. In glioma treatment, first-generation photosensitizers were tested in clinical trials already in the 1980s. These include hematoporphyrin derivatives (HpD), a porfimer sodium, and dihematoporphyrin ether (DHE), which have been used to treat other oncologic diseases. Small, heterogeneous cohorts with case numbers ranging from 2 to 80 patients were included. Overall, an improvement in progression-free survival and overall survival was reported compared with standard treatment of patients with malignant gliomas. Nevertheless, first-generation photosensitizers do not selectively accumulate in glioblastoma tissue, resulting in a higher rate of side effects, e.g., in the development of extensive perifocal edema. Therefore, the so-called second-generation photosensitizers with increased ROS production and improved selectivity for tumor tissue were used. These include porphyrin or chlorin-based molecules or precursors, such as 5-aminolevulinic acid (5-ALA), talaporfin sodium, boronated porphyrins, temoporfin, and benzoporphyrin derivatives. Since the approval of 5-ALA for intraoperative visualization of malignant gliomas by the European Medicine’s Agency (EMA) in 2007 and the US Food and Drug Administration (FDA) in 2017, preference has been given to this agent as a photosensitizer in glioma surgery and therapy. 5-ALA is a natural precursor of heme and is converted to protoporphyrin IX in mitochondria by several enzymatic steps. In the tumor tissue, there is an accumulation of PPIX and a release of ROS and radicals after exposure to light with a wavelength of 630–635 nm. Due to the selectivity of ALA for tumor tissue and rapid elimination, the spectrum of side effects is limited. Since 2000, research has been conducted on third-generation photosensitizers under in vitro conditions. These are photosensitizer-loaded nanocarriers that exhibit high local selectivity and can transport the photosensitizer directly to tumor tissue while simultaneously transporting exogenous oxygen or chemotherapeutic agents [9, 15].

Currently, there are several clinical trials investigating the efficacy of PDT using 5-ALA. These are three phase II trials: stereotactic photodynamic therapy with 5-aminolevulinic acid (Gliolan) in recurrent glioblastoma (NCT04469699), PD L 506 for stereotactic interstitial photodynamic therapy of newly diagnosed supratentorial IDH wild-type glioblastoma (NCT03897491), and dose finding for intraoperative photodynamic therapy of glioblastoma (NCT04391062).

Cramer et al. [9] conducted a meta-analysis including data on almost more than 1000 patients with first diagnosis or recurrence of glioblastoma treated with photodynamic therapy. The median overall survival for patients with initial diagnosis was 16.1 months and for patients with recurrence 10.3 months. Clinical trials showed prolonged progression-free survival and overall survival compared to control groups with standard therapy. The included patients comprise approximately 600 patients with a first diagnosis, while about 270 patients had a recurrent glioblastoma. Overall, different photosensitizers (talaporfin sodium, 5-ALA, porfimer sodium, hematoporphyrin derivative, temoporfin, boronated porphyrin) as well as wavelengths (628 to 664 nm) and energy density (range, 8 to 400 J/cm^2^) were used. Due to the heterogeneity of the data, estimation of efficacy of PDT is limited. Four clinical trials investigated the use of 5-ALA as a photosensitizer in PDT (Table 1).

**Table 1:** Clinical trials using 5-ALA PDT

Author (date)	Number of patients	Outcome measure (months)
Beck et al. (2007)	10 (recurrent GBM)	Median OS 15
Johansson et al. (2013)	5 (4 recurrent GBM)	PFS 3, 9, 29, 30, 36
Schwartz et al. (2015)	15	Median PFS, 16; 3-year survival, 56%
Eljamel et al. (2008)	13	Median OS 13.2

Beck et al. [3] conducted a phase 1 pilot study with interstitial 5-ALA-PDT in ten patients with recurrent glioblastoma and demonstrated a median prolonged survival of 15 months in patients after PDT compared to patients with standard treatment. Similarly, in five patients with non-resectable glioblastoma, Johansson et al. [17] were able to demonstrate progression-free survival of 29 to 36 months in three patients after PDT with 5-ALA. A significant prolongation of progression-free survival (median PFS, 16 vs 10.2 months, *p* < 0.001; 3-year survival, 56% vs 21%) in non-resectable glioblastomas with a patient group of 112 patients who received complete tumor resection and adjuvant therapy according to the EORTC protocol was confirmed by Schwartz and colleagues [31]. Eljamel et al. [11] used Photofrin as a photosensitizer for PDT next to 5-ALA and observed an overall survival of 13.2 months for the 13 patients in the study group compared to an overall survival of 6.2 months in the control group consisting of 14 patients with standard therapy for glioblastoma.

## Sonodynamic therapy (SDT)

Similar to PDT, sonosensitizers are stimulated with low or high intensity ultrasound causing an effect called sonoluminescence, which leads to the release of ROS and the induction of tumor cell death through oxidative stress, DNA damage, and apoptosis. Furthermore, the cytotoxic effect presumed to be additionally based on the generation of hyperthermia and the cavitation effect. In this process, microbubbles in the resection cavity are caused to oscillate by the ultrasound-induced pressure, resulting in mechanical lesions of the surrounding tissue and the release of hydroxyl radicals and ROS [16]. Similarly, an immunomodulatory effect as well as an antiangiogenic effect by inhibiting proliferation and migration of endothelial cells has been described in in vitro studies [13, 45]. Intensities of sonication of 0.2 to 25 W/cm^2^ were applied, and frequencies of 0.5 to 3 MHz were used. The duration of the intervention was 10 ms to 20 min. According to the known photosensitizers used for PDT, the following sonosensitizers have been analyzed: 5-ALA, fluorescein (FL), sinoporphyrin sodium (DVDMS), hematoporphyrin monomethyl ether (HMME), temozolomide (TMZ), and Photofrin [4]. Among these, 5-ALA is the most extensively studied sonosensitizer whose biocompatibility and biosafety have been widely demonstrated in clinical studies [7]. An advantage over PDT is the greater depth of penetration of the focused ultrasound into the tissue, so that deep-seated and diffuse tumors can be reached and treated more efficiently. In several in vitro studies, efficacy of SDT was demonstrated via apoptotic effects and generation of ROS. Similar results have been shown by in vivo studies in animal models [4, 24, 33, 38, 39].

There are four ongoing phase 0 and phase 1 clinical trials: sonodynamic therapy with exablate system in glioblastoma patients (NCT04845919), study of sonodynamic therapy using sonala-001 and exablate 4000 type 2 in recurrent GB (NCT05370508), study to evaluate 5-ALA combined with CV01 delivery of ultrasound in recurrent high-grade glioma (NCT05362409), and study of sonodynamic therapy in participants with recurrent high-grade glioma (NCT04559685). Whereas the exablate system delivers high energy focused ultrasound to small volumes, CV01 delivers ultrasound below the upper threshold defined by FDA for diagnostic ultrasound and is less focused. Ultimately, if both proven to be of efficacy, combination of focused and more diffuse ultrasound may be prove to be synergistic. Sonodynamic therapy is furthermore an interesting mode to explore, since in theory, this therapy can be repeated as often as necessary, since repeat applications of 5-ALA have not been observed to result in additive toxicity [30] and it is unlikely that repeated ultrasound will cause cumulative damage to non-tumor tissue. The latter assumption, however, requires more clinical tests.

## Radiodynamic therapy (RDT)

Radiodynamic therapy is based on an ionizing radiation-induced excitation of certain photosensitizers or radioluminophores coupled with photosensitizers. Like the mechanism of action of PDT, the formation of ROS is induced, which causes antitumor effect. However, in this type of therapy, the effect can be extended to deeper tissue layers, due to the penetrating power of photon beams [41]. The radiosensitizer is applied before every single irradiation fraction according to its half-life. With repetitive administration, there is a risk of accumulation of the substance with an increased risk of developing side effects, leading to a debate in terms of biosafety [40]. Some in vitro and in vivo studies including clinical trials have investigated the efficacy of different radiosensitizing agents in the treatment of malignant gliomas: nitroimidazoles, nicotinamide and carbogen, tipifarnib, efaproxiral, tirapazamine, halogenated bromodeoxyuridine and iododeoxyuridine, poly (ADP-ribose) polymerase proteins, motexafin gadolinium, difluoromethylornithine, interferon-alpha-2a, lovastatin, and 5-ALA [26].

The following ongoing clinical trials address the efficacy of radiosensitizing agents: Phase I/II Dose Escalation Trial of Radiodynamic Therapy (RDT) With 5-Aminolevulinic Acid in Patients With First Recurrence of Glioblastoma (NCT05590689), Phase I/II Trial to Assess the Radiosensitizing Effect of ZARNESTRA in Patients With Glioblastoma Multiforme (NCT00209989), Phase 0/I Clinical Trial of the ATM-Inhibitor WSD0628 in Combination With Radiation Therapy for Recurrent Brain Tumors (NCT05917145).

## Conclusions and future perspectives

In recent years, the use of external stimuli-responsive therapies has moved into the focus of local cancer therapy and has thus become relevant for glioblastoma treatment. Clinical studies have already demonstrated the effectiveness of these types of therapy at low levels of toxicity. The use of PPIX-based therapies is in particular extensively investigated in preclinical and clinical studies. Prospective controlled clinical trials are currently recruiting. With the prospect of targeted and individualized oncological therapy, external stimuli-responsive therapies will gain in relevance and importance in the future. While research is currently focused on gliomas, other tumors which accumulate PPIX might also become targets, even if they are not considered surgical entities, such as CNS lymphomas, which strongly convert 5-ALA to PPIX [12].

## Abbreviations

ALA: Aminolevulinic
DHE: Dihematoporphyrin ether
EMA: European Medicines Agency
EORTC: European Organisation for Research and Treatment of Cancer
FDA: Food and Drug Administration
HpD: Hematoporphyrin derivatives
NCT: National Center for Tumor Diseases
PDT: Photodynamic therapy
PPIX: Protoporphyrin IX
RDT: Radiodynamic therapy
ROS: Reactive oxygen species
SDT: Sonodynamic therapy
